# The Potential of Near-Infrared Spectroscopy as a Therapeutic Tool Following a Stroke (Review)

**DOI:** 10.17691/stm2025.17.2.07

**Published:** 2025-04-30

**Authors:** O.A. Mokienko

**Affiliations:** Senior Researcher, Mathematical Neurobiology of Learning Laboratory; Institute of Higher Nervous Activity and Neurophysiology of the Russian Academy of Sciences, 5A Butlerova St., Moscow, 117485, Russia; Senior Researcher, Engineering Center; N.I. Pirogov Russian National Research Medical University, 1 Ostrovityanova St., Moscow, 117997, Russia; Researcher, Brain–Computer Interface Group of Institute for Neurorehabilitation and Restorative Technologies; Research Center of Neurology, 80 Volokolamskoye Shosse, Moscow, 125367, Russia

**Keywords:** near-infrared spectroscopy, neuroimaging, stroke, neurological rehabilitation, neurofeedback, neuromodulation

## Abstract

The advancement of novel technologies for the rehabilitation of post-stroke patients represents a significant challenge for a range of interdisciplinary fields. Near-infrared spectroscopy (NIRS) is an optical neuroimaging technique based on recording local hemodynamic changes at the cerebral cortex level. The technology is typically employed in post-stroke patients for diagnostic purposes, including the assessment of neuroplastic processes accompanying therapy, the study of hemispheric asymmetry, and the examination of functional brain networks. However, functional NIRS can also be used for therapeutic purposes, including the provision of biofeedback during rehabilitation tasks, as well as the navigation method during transcranial stimulation. The effectiveness of therapeutic NIRS application in stroke patients remains insufficiently studied, despite existing scientific evidence confirming its promising potential as a treatment method.

The review examines the published literature on the therapeutic applications of NIRS after stroke, evaluating its potential role in the rehabilitation process. The paper describes NIRS features, advantages, and disadvantages, determining its position among other neuroimaging technologies; analyzes the findings of neurophysiological studies, which justified the clinical trials of NIRS technology; and evaluates the results of the studies on the therapeutic use of NIRS in post-stroke patients. Two potential applications of NIRS for therapeutic purposes following a stroke were suggested: the first was to provide real-time feedback during movement training (motor or ideomotor ones, including that in brain–computer interface circuits), and the second was to facilitate navigation during transcranial stimulation.

Based on a comprehensive literature review, there were proposed and justified further research lines and development in this field.

## Introduction

Stroke remains one of the major challenges of public health. According to the statistics, in 2019, stroke ranked third in the world among other causes of mortality and disability of population at a global scale. It is responsible for 5.7% (the range 5.1–6.2%) of the total life years lost due to different diseases. Stroke incidence dynamics is of greater concern. Over the period from 1990 to 2019, there was a considerable increase of primary cases of the disease — by 70.0% (variations 67.0–73.0%) [1]. In Russia in recent years there have been registered from 430,000 to 470,000 stroke cases annually; during a year following a stroke 95,500 people completely cease their work activities, and over the half of those who survived require assistance and care from others [2]. At every stage of medical rehabilitation, there is the lack of the staff to provide physical therapy (basic methods of therapeutic exercises) [3], therefore, the development of available rehabilitation technologies is still much- needed. Innovative technologies can compensate to different extents for the intensity of physical therapy insufficient for functional recovery and be used when there are some constraints in providing therapeutic exercises or accompany the therapy at various stages of medical rehabilitation [4–7].

Near-infrared spectroscopy (NIRS) is a noninvasive optical imaging technique that records the changes in the concentrations of certain hemoglobin fractions of microcirculation up to 3 cm deep from skin covering [8, 9]. Functional NIRS (fNIRS) similarly to functional magnetic resonance imaging (fMRI) enables to record the cerebral cortex activity by measuring the local oxygenation dynamics, and at the same time it being portable, resistant to electromagnetic interferences, and motion artifacts, and much cheaper technology [10–12].

Currently, fNIRS is widely used in clinicalbased studies in post-stroke patients to assess neurophysiological characteristics of rehabilitation efficiency [13–17] and recovery prognosis [18, 19], to study recovery mechanisms [20, 21], hemispheric relation and asymmetry [21–25]. However, fNIRS can also be employed as a therapeutic technology for training with biological feedback (BFB), in braincomputer interface (BCI) and for functional navigation when using neuromodulation methods [9, 26, 27].

In contrast to diagnostic strategies of fNIRS use, its therapeutic ones are underexplored.

**The present review aimed at** analyzing the published data on the therapeutic application of fNIRS after stroke to determine a possible position of the technology in the rehabilitation process and ground further research lines and development in the sphere.

## Literature source searching methodology

To search the review articles devoted to NIRS used after stroke and published within the recent 5 years in PubMed/MEDLINE the following enquiry was used: (near-infrared spectroscopy [tiab] OR NIRS [tiab]) AND (stroke [mh] OR stroke [tiab]) AND (meta-analysis [pt] OR review [pt] OR systematic review [pt]) AND 2020:2024 [dp]. The searching date is June 10, 2024.

To search literature sources concerning clinical research of using NIRS following a stroke in PubMed/MEDLINE the following enquiry was used: (near-infrared spectroscopy [tiab] OR NIRS [tiab]) AND (stroke [mh] OR stroke [tiab]) AND (clinical trial [pt] OR randomized controlled trial [pt]). The searching date is June 17, 2024.

Available Russian publications were searched in eLIBRARY.RU by key words “NIRS” and “near infrared spectroscopy”. The searching date is July 5, 2024.

Additionally, clinical trial protocols were searched at https://clinicaltrials.gov by key words “near-infrared spectroscopy” and “stroke”. The searching date is July 8, 2024.

## Features, advantages, and disadvantages of functional near-infrared spectroscopy

NIRS technique enables to record the changes in hemoglobin concentration in the microcirculation (in the vessels with a diameter of less than 1 mm) at a depth of up to 3 cm from the head surface. For this purpose, it has been used in biomedical studies since the late 1970s [28]. It is based on the light ability in nearinfrared range to penetrate biological tissues, where it is absorbed and mainly scattered. Radiation attenuation in the range 650–1000 nm is primarily due to its absorption by hemoglobin [28–30].

fNIRS systems utilize the light sources with an optic window from 650 to 1000 nm, light detectors, as well as flexible fiber optics. Moreover, fiber optics are suitable for any head position and do not require study subject immobilization. Sources and detectors are primarily fixed in an elastic cap at a distance of 1.5–5.0 cm (more frequently — 3 cm) from one another. A signal recording channel includes a pair: emitter–detector. Based on the information on incident and outgoing light and applying a modified Bouguer–Lambert–Beer law, light attenuation is calculated. This enables realtime measurement of oxygenated hemoglobin (HbO), deoxygenated hemoglobin (HbR), and total hemoglobin (HbT) concentrations [28, 29, 31]. Current NIRS systems can contain up to several hundreds of channels with time resolution up to 250 Hz and space resolution up to 10 mm [8, 28].

fNIRS performing is based on the following processes accompanying the brain cortex activation. The increase in cortical neuron activity results in two effects: 1) neurometabolic consisting in HbO concentration decrease and HbR concentration increase due to increased tissue breathing; 2) hemodynamic, which, in contrast, leads to HbO concentration increase and HbR concentration decrease due to local blood flow increase. Since a hemodynamic effect significantly outweighs the metabolic one, the markers of local cortical activation during fNIRS include the increase in HbO concentration and the decrease in HbR concentration. The increase in HbO concentration also results in HbT concentration growth (see the Figure) [8, 9].

**Figure:**
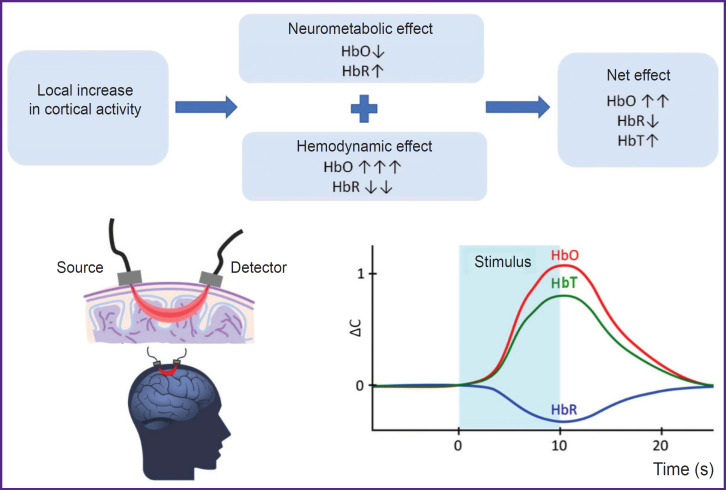
Signal origin and local cortical activation markers in performing functional nearinfrared spectroscopy (adapted from [8] and [9]) HbO — oxygenated hemoglobin, HbR — reduced hemoglobin, HbT — total hemoglobin, ΔС — concentration change

As a neuroimaging tool, fNIRS offers the following advantages: ease of administration (requires neither electrode gel application nor the study subject immobilization); portability; high movement interference resistance; electromagnetic compatibility; enhanced signal reliability (enabled by simultaneous monitoring of multiple parameters: HbO, HbR, and HbT concentrations); relatively low cost [8, 28]. Due to its portability and resistance to motion artifacts, fNIRS — unlike fMRI — allows for the performance of functional tasks in seated or standing positions. Owing to the mentioned advantages, fNIRS can be employed in a number of situations where fMRI is impractical: in children [32–34], in people with metal implants, those suffering from cognitive disorders, claustrophobia, and other conditions [9, 35]. The feasibility of using NIRS in home settings after stroke has been demonstrated [36]. In applications where fNIRS can serve as an alternative to electroencephalography (EEG) or fMRI, for example, for BFB, the technique is a compromise solution between good time resolution of EEG and good space resolution of fMRI [9]. The main fNIRS disadvantages are the following: limited imaging depth (hemodynamic response can be assessed at a cortical level, but not in deep brain structures), relatively low spatial resolution, temporal signal delay caused by the slow nature of hemodynamic response, signal interference from the patient’s body temperature fluctuations and cardiac activity (e.g., heartbeat). Furthermore, current NIRS technologies are restricted to measuring relative changes in hemodynamic parameters rather than providing absolute quantitative values [8, 9, 27, 31].

## Neurofeedback control by NIRS signal

NIRS can be used for feedback during motor imagery, i.e., the mental reconstruction of the sense of motion [37–41]. The motor imagery is accompanied by the activation of brain structures involved in the process of a real movement. It is supposed to have a positive impact on neuroplasticity, which underlies the motor recovery [42]. It is worth noting that motor imagery training (ideomotor training) is possible even in severe limb paresis, when active motor exercises are limited. A positive effect of ideomotor trainings on motor function recovery after stroke was demonstrated in many studies [43, 44]. Biofeedback enables to control a patient performing a mental task of the motor imagery improving the efficiency of such training.

An ideomotor training with feedback using NIRS involves the following stages. An operator (a patient or a healthy volunteer) takes an instruction to imagine certain limb movement; and the mental activity is accompanied by the changes in HbO and HbR concentrations in the cortical areas responsible for the movement, it being registered by NIRS; then NIRS signal is recognized by computer programs and converted into a signal for feedback. The feedback is delivered to the operator (in its easiest form — as a visual signal on a computer monitor) [37–41]. The most popular form of such approach implementation is the use of BCI technology [45–47].

According to the latest definition, BCI is a system that measures the brain activity and converts it (approximately) in real-time into functionally useful output signals for replacement, recovery, enhancement, supplement, and/or improvement of natural output brain signals, by that changing the current interaction processes between the brain and its external or internal environment [48]. EEG-based BCI is used for motor imagery trainings after stroke, and a signal of the brain activation, as a rule, is the desynchronization of the sensorimotor rhythm. Despite a number of metaanalyses have demonstrated EEG–BCI efficiency after stroke, the main issues of the technology implementation are the requirement to apply electrode gel to the patient’s scalp and its low resistance to interference [49].

The feasibility of NIRS–BCI application as an alternative to EEG–BCI to perform ideomotor trainings was shown in the early 2000s in healthy volunteers [37–39]. Later, some controlled studies with healthy volunteers stated real rather than fictitious feedback by NIRS signal to induce specific and focused activation of the motor cortex during movement imaging [50–52], and the course of such trainings was accompanied by improved agility of hand movement [52]. Moreover, repetitive trainings with NIRS feedback in healthy people were demonstrated to improve the ability to imagine movements both according to the assessment of the subjects themselves [50] and according to neuroimaging data (there was the increased specificity of motor cortex activation) [50, 51].

The healthy volunteers in the study [53] were offered to control the activity of the supplementary motor cortex with BFB using NIRS signal or a fictitious BFB. However, there were specified none certain strategies to control signals, e.g. motor imagery strategies. Finally, only a group with real feedback was observed to have increased activity of the supplementary motor cortex and improved postural balance but not the agility of hand movement.

The study [54] investigated a hybrid BCI based on EEG and NIRS recording during the movement imaging by healthy volunteers. The brain activity signals were converted into the control over functional electric stimulation (FES) of the hand muscles. In the control group, FES was triggered randomly, not by BCI signals. As a result of trainings, the BCI group, compared with the control one, had more expressed cortical activation according to EEG and NIRS parameters.

The findings of the studies with healthy volunteers provided justification of carrying out several clinical trials of using feedback by NIRS signal in post-stroke patients [55–60], two of the studies were randomized controlled trials (RCT) (see the Table).

**Table:** The studies on NIRS application to present feedback in motor post-stroke rehabilitation

Source	Study design and therapy regimen	Patients	Result
Mihara et al., 2013 [55]	RCT: hand motor imagery with feedback from premotor cortex (intervention) or with occasional feedback (control) - 6 sessions for 20 min within 2 weeks	Subcortical stroke with onset more than 12 weeks ago, n=20	Increase in the FMA score by 6.6 in the intervention group and by 4.2 in the control group (p<0.001); the correlation of cortical activity changes with motor recovery (r=0.61; p<0.05)
Rieke et al., 2020 [56]	Clinical case: wrist extension trainings for 3 days with fMRI sessions (162 movements per session), then for 4 weeks - 10 sessions (144 movements per session) of NIRS-FES control	Subcortical hemorrhagic stroke with onset 8 years ago, n=1	Increase in the FMA-UL score by 10 scores; active wrist extension increase by 18°
Mihara et al., 2021 [57]	RCT: movement and balance imaging with feedback from premotor cortex (intervention), or with occasional feedback (control) - 6 sessions for 10 min within 2 weeks	Subcortical hemorrhagic stroke with onset more than 12 weeks ago, n=54	Reduced timed 'up and go' test by 12.8 s in the intervention group and by 5.5 s in the control group (p<0.05); increase in Berg balance scale by 3.2 scores in the therapy group, and by 1.5 scores in the control group (p<0.001)
Lyukmanov et al., 2023 [58] Isaev et al., 2024 [59] Mokienko et al., 2024 [60]	With historical control: hand motor imagery with visual feedback using NIRS-BCI - 10 sessions within 2 weeks, control - EEG-BCI from the previous study	Cortical ischemic stroke with onset from 1 day ago, n=15 (NIRS-BCI), n=17 (EEG-BCI)	Increase in the ARAT and FMA-UL scores on average by 5.0 scores (p<0.05); average and maximum achieved control accuracy for NIRS-BCI were higher than for EEG-BCI (p<0.05)

N o t e. In the mentioned studies, the trainings using NIRS was applied in addition to a standard rehabilitation program. ARAT — Action Research Arm Test, NIRS — near-infrared spectroscopy, BCI — brain–computer interface, RCT — randomized controlled trial, fMRI — functional magnetic resonance imaging, FES — functional electric (muscular) stimulation, FMA-UL — Fugl–Meyer assessment for an upper limb.

RCTs have demonstrated the advantage of ideomotor trainings with NIRS feedback for the recovery of hand movement [55], balance, and walking [57]. In both, studies the use of real but not fictitious feedback was accompanied by significant activation of motor associative cortex (premotor, supplementary motor).

The NIRS–BCI technology in a more complete implementation was used in the study [56], which describes the results of controlling functional electrical stimulation of muscles using brain NIRS signals in a clinical case. A patient first had three fMRI-feedback sessions to determine the cerebral cortex areas activated in wrist extension. It was taken into consideration when choosing NIRS channels location. During trainings with BCI, the patient attempted to straighten his wrist, the brain NIRS signals being converted into the control commands of wrist muscular FES to facilitate movements. Despite the neurophysiological ground of the approach and the impressive result of the hand motor recovery following 8 years after stroke, in this case, NIRS–BCI–FES technology contribution is unclear, since each training itself included more than 140 movement repetitions.

The studies [58–60] using NIRS–BCI involved the patients imagined the hand motor task from Action Research Arm Test (ARAT), which performing was the most difficult due to the post-stroke paresis. The results of the hand movement recovery and BCI control quality were higher when using NIRS–BCI compared to EEG– BCI. The authors explained it by the fact that the cortical activity indicators in NIRS are several parameters (HbO, HbR concentration measurement) that facilitates the task of the BCI classifier. The second explanation was the grater interference immunity of NIRS compared to EEG [60].

## Navigation through near-infrared for neurostimulation

Rhythmic transcranial magnetic stimulation (rTMS) is a non-invasive brain stimulation method that modulates the excitability of the target cortical zone. According to current views, rTMS effect mechanisms are based on the induction of long-term potentiation or long-term depression [61]. TMS effects may result from either neuromodulation of the stimulated brain regions or changes in functional brain networks connecting several remote areas [62–64].

Numerous systematic reviews and meta-analyses described the efficiency of the approach in post-stroke patients in relation to recovery of motor function [63, 65], cognitive function [66, 67], speaking [68], swallowing [69], treatment of post-stroke central pain syndrome [70, 71], or post-stroke depression [72].

Despite widespread application of rTMS in poststroke patients, the search for optimal parameters and the development of strategies to select individualized rTMS protocols remain ongoing [61, 64]. Until now the mechanisms of TMS effect on target cortex have been underexplored, and it is unclear how the local effect is spreading in the central nervous system. Moreover, the problem of choosing stimulation points has no solution so far. In stimulating motor areas, the predominant approach includes the location determination of the point with the highest amplitude of motor evoked potentials. However, this approach often does not work due to high individual excitability threshold, presence of stroke focal focus, or due to other unstudied reasons. Therefore, some alternative rTMS navigation methods are needed [62, 73].

Optically measured NIRS signals are not susceptible to electromagnetic interference, and the technology does not affect the magnetic properties of TMS coil that enables to use these approaches simultaneously. Conducting NIRS during rTMS enables to make an objective quantitative assessment of neurophysiological responses during stimulation in both spatial and time coordinates. This makes it possible, including in realtime mode, to adjust the parameters of rTMS and select stimulation targets [74, 75].

In the study [76], the patients with aphasia resulting from stroke with an onset of more than 12 months ago underwent the course of 10 sessions of therapeutic rTMS in addition to intensive speech therapy. Before the course of therapy using NIRS, the cortical activation areas were determined during the performance of a speech task. Based on NIRS findings, the rTMS lateralization and mode were determined. There was observed a pronounced therapeutic effect, however, the study involved no control group, and the sample included 8 patients only. Thus, the authors proposed a personalized approach for choosing rTMS protocol based on individual neuroimaging using NIRS.

The double-blind RCT involving patients with poststroke arm paresis compared the protocols of rTMS navigation using NIRS and motor evoked potentials [77]. The points for rTMS were determined in all cases when NIRS was used as navigation, even when the motor evoked responses could not be recorded. After a 10-day therapy course, both groups, unlike the sham stimulation group, showed improvement in upper extremity motor function based on two assessment scales. However, improvement in elbow joint movements was observed only in the NIRS navigation group. These findings highlight the feasibility of combining rTMS and NIRS technologies to restore motor function after stroke.

## Further lines of research and developments

Taking into consideration the importance of poststroke rehabilitation, unique fNIRS characteristics, and the open scientific practical issues of using neuromodulation methods, supplementary research of the therapeutic application of the method remain in demand. To compare the clinical efficiency of NIRS– BCI with more studied, though less convenient EEG– BCI technology, it is necessary to carry out prospective comparative studies in parallel groups. Moreover, further research can also use NIRS–BCI with feedback through the exoskeleton of upper [78, 79] or lower [80] limbs. However, FES can become the most physiological and effective feedback in BCI [81–84].

Motor imagery trainings, in addition to motor recovery, can contribute to the improvement of cognitive functions and cognitive components of a motor process (e.g., motion planning) [85, 86]. Therefore, it is necessary to pay attention to studying the efficiency of NIRS–BCI trainings in relation to recovering cognitive functions in post-stroke patients.

One of the novel applications of feedback by NIRS signal can be the recovery of swallowing. Several studies involving healthy volunteers managed to achieve the successful regulation of motor cortex activity (inferior frontal gyrus) associated with swallowing using feedback through NIRS [87–89].

An important practical aspect is further cost-cutting of NIRS technology and its simplification for patient’s selfuse. There have already been published several clinical trial protocols of NIRS-based developments to perform motor and mental trainings at home [90, 91].

Open public access to structured datasets of brain NIRS signal recordings will accelerate the improvement of algorithms for their processing and classification. By now, there have been published several NIRS data sets obtained from healthy people [92–95], and just one — from post-stroke patients [59].

The hybrid EEG–NIRS BCI is of great interest for brain-machine interface developers [54, 96–99]. The combination of two methods of signal recording will enable to improve BCI control quality, however, hybrid BCI appears to be less convenient for clinical practice.

In respect to any neurorehabilitation techniques, it is important to understand the underlying models of motor control and learning [100]. Currently, there remains the understudied issue — the activity of which cortical areas and in what succession is appropriate to modulate using feedback to achieve more pronounced functional recovery [9, 27]. A number of carried out studies showed biofeedback using NIRS to be performed by signals from associative motor areas, which may be involved in movement suppression. Therefore, supplementary investigations are needed to justify the selection of the brain signal sources. Moreover, it is highly interesting to set up the feedback based on the connectivity of a functional network of several areas rather than the signals from certain areas [9].

The results of two clinical studies of rTMS navigation using NIRS have been described, and their protocols have been published [101, 102]. However, despite the existing theoretical prerequisites for further clinical studies, this direction is developing rather slowly. The technical difficulties related to the application of hybrid NIRS–TMS systems (or NIRS in combination with transcranial electrical stimulation [103, 104]) should not prevent from carrying out research in this field, since they can improve understanding of fundamental aspects of motor recovery after stroke.

## Conclusion

Thus, by now there have been suggested two trends of fNIRS application for therapy after stroke: to present feedback during movement trainings (motor or ideomotor, including those in BCI contour) and for navigation when performing transcranial stimulation.

Despite the long-term existence of NIRS technology, in post-stroke rehabilitation it has been primarily studied as a diagnostic rather than therapeutic tool.

Having a number of shortcomings, NIRS exhibits certain advantages over some alternative approaches of feedback presenting during movement trainings or rTMS navigation methods. Increasing technology availability, including through the emergence of new commercial products, as well as additional well-designed clinical trials, will better justify fNIRS place in post-stroke rehabilitation protocols.
